# ISAK-Based Anthropometric Standards for Elite Male and Female Soccer Players

**DOI:** 10.3390/sports12030069

**Published:** 2024-02-23

**Authors:** Cristian Petri, Francesco Campa, Francis Holway, Luca Pengue, Luis Suarez Arrones

**Affiliations:** 1 Section of Physical Education and Sport, Department of Sport and Informatics, Pablo de Olavide University, 41013 Sevilla, Spain; cpet2@alu.upo.es (C.P.);; 2 A.C.F. Fiorentina S.r.l., 50137 Florence, Italy; lpengue@acffiorentina.it; 3 Department of Biomedical Sciences, University of Padua, 35131 Padova, Italy; 4 Medical Department of Hindu Rugby Club, Don Torcuato 600-698, Argentina; fholway@gmail.com

**Keywords:** anthropometry, adipose tissue, body composition, morphology, reference percentiles, somatotype, skinfolds, Z-scores

## Abstract

This study aimed to provide reference values for anthropometric characteristics of elite male and female soccer players, considering a group of individuals from the general population as controls. The anthropometric profiles of 357 elite soccer players [184 males (age 24.3 ± 4.3 y) and 173 females (age 25.2 ± 5.1 y)] participating in the first Italian league (Serie A) and 363 subjects from the general population [188 males (age 24.2 ± 4.8 y) and 175 females (age 25.0 ± 5.1 y)] were measured according to the guidelines of the International Society for the Advancement of Kinanthropometry (ISAK). Reference percentiles for stature, body mass, circumferences, eight skinfolds (biceps, triceps, subscapular, suprailiac, supraspinal, abdominal, front thigh, and calf), breadths, and somatotype were calculated and stratified by player position and sex. No difference (*p* > 0.05) was found in age between the two groups. Soccer players showed lower values for the sum of the eight ISAK skinfolds than individuals from the general population of the same sex. This suggests lower adipose tissue, as indicated by a lower endomorphic component. The somatotype was endomorphic mesomorph and mesomorphic endomorph for the male and female individuals from the general population, respectively. The male soccer players were ectomorphic mesomorphs, while the females were balanced mesomorphs, defining a sport-specific morphology. This study provides sex- and role-specific anthropometric standards for elite soccer players. Raw anthropometric reference values may be useful for evaluating body composition without using any predictive equations or assumptions.

## 1. Introduction

Soccer, commonly known as football outside North America, is a team sport played between two teams of eleven players each. Serie A is the foremost professional soccer league in Italy, where each team typically maintains approximately 25–30 players. With 20 teams participating in the league, the total number of players involved ranges approximately between 500 and 600. This dynamic sport necessitates a multifaceted skill set encompassing precision, agility, and collaborative synergy. Beyond the evident athletic requisites, the intrinsic physiological demands that soccer places on professional players exert profound effects on the body composition of the practitioners [1]. The exigencies of the game, coupled with stringent training regimens, bear consequential implications for variables such as fat and muscle mass, endurance, and overall physical fitness, underscoring the intricate interplay between the sport’s demands and the physiological adaptations exhibited by its elite athletes [2,3]. A soccer team comprises various positions, including forwards, midfielders, defenders, and goalkeepers. For example, forwards and wingers typically undergo intense bursts of speed and agility, requiring explosive power and quick acceleration to penetrate opposing defenses. Midfielders, on the other hand, often cover extensive distances during a match, necessitating exceptional endurance and stamina to contribute both defensively and offensively. Defenders focus on strength, agility, and spatial awareness to thwart opposing attackers and initiate build-up play. Goalkeepers require agility, reflexes, and explosive movements to make critical saves. These distinct roles may contribute to differences in players’ body composition and physical attributes due to the specialized movements and performance expectations associated with their positions [4].

In soccer, assessing body composition is of paramount importance, as it proves instrumental in monitoring the effects of diet and training throughout the competitive season, as well as during phases of detraining [5]. Surface anthropometry is a rapid and cost-effective technique for assessing body composition, providing insights into qualities related to fat, muscle, and skeletal components [6]. Currently, the International Society for the Advancement of Kinanthropometry (ISAK) measurement standards serve as standardized guidelines for the assessment of anthropometric parameters [6]. This method involves measuring various surface body dimensions, such as skinfolds and circumferences, to derive information about an individual’s morphological characteristics. Surface anthropometry serves as a valuable tool in understanding the morphological aspects of body composition, offering practical applications in fields ranging from sports science to clinical assessments. Indeed, body circumferences provide insights into body geometries and are influenced by both muscle and adipose tissue, particularly affecting endurance performance and movement quality [7,8]. Skinfolds, on the other hand, yield highly informative parameters for subcutaneous adipose tissue, a factor that negatively impacts endurance performance and mobility [2,4]. Meanwhile, somatotype allows an investigation into the morphological features studied by research to gather information about the athlete’s dimensions and proportions [9]. 

To attain a valid body composition assessment, having reference values is crucial, especially for raw parameters that remain unaffected by estimation errors. Reference values serve as benchmarks or standards against which an individual’s body composition can be compared. Unlike estimates derived from predictive equations, raw anthropometric measures allow a more direct and reliable assessment, offering a baseline for understanding body composition features [10]. Indeed, studies providing reference parameters for quantitative estimates of body mass components are only applicable if the same procedures, such as the predictive equations, are applied. Currently, these predictive equations are not available on an anthropometric basis for soccer players. For this reason, reference data for raw anthropometric parameters are preferable. By utilizing such reference values, practitioners can tailor training and nutrition plans to individual athletes, considering the unique demands associated with their sex and specific role in the game [10]. This nuanced approach contributes to a more targeted and effective optimization of performance and overall well-being.

Currently, to our knowledge, there are no specific anthropometric reference values available for elite soccer players. This absence of targeted data may represent a significant gap in the analysis of body composition and specific physical characteristics among those involved in professional soccer. Therefore, the objective of this study was to select elite male and female soccer players and compare them to a group of individuals of the same age and gender from the general population. The aim was to delineate the anthropometric standards of soccer players, further categorizing them based on playing positions. Our hypothesis was that soccer players of both sexes would exhibit anthropometric characteristics different from those of the general population of the same age, necessitating the need for specific reference values.

## 2. Materials and Methods

### 2.1. Participants and Study Design

A total of 357 elite soccer players [184 males (age 24.3 ± 4.3 y) and 173 females (age 25.2 ± 5.1 y)] from the first Italian division (Serie A) and 363 subjects from the general population [188 males (age 24.2 ± 4.8 y) and 175 females (age 25.0 ± 5.1 y)] living in Italian territory were included in this observational comparative study. Data collection was conducted during the first half of their competitive season during a national gathering. Testing procedures were performed for each player in the morning, the day before the main weekly match. After collecting the measures from the soccer players, the same number of subjects stratified for age and sex were recruited from the general population and used as a control group. The following inclusion criteria were used: (i) participation in the first Italian league for soccer players and no professional sport involvement for at least 2 years for individuals in the general population, (ii) negative test outcomes for performance-enhancing drugs, and (iii) freedom from current medication use and injuries at the time of the test. The participants provided a signed informed consent, and the study procedures were approved by the local Ethic Committee (code: HECDSB22023), attesting to the fulfillment of all human research standards set out by the Declaration of Helsinki.

### 2.2. Procedures 

**Figure 1:**
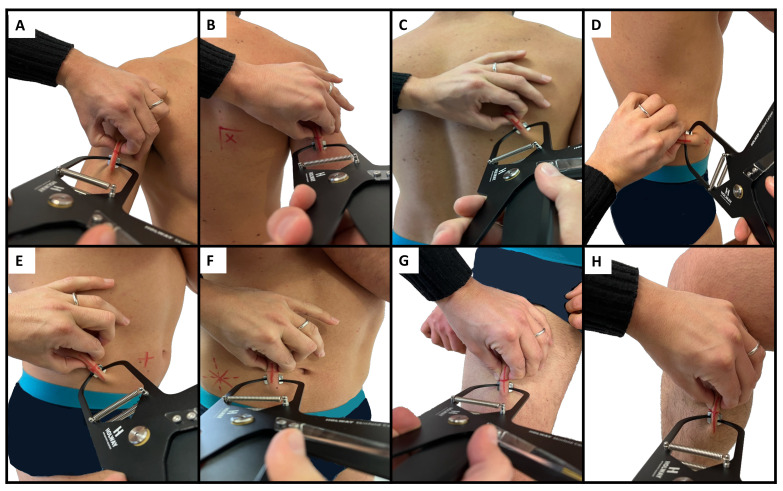
Illustration of the biceps (**A**), triceps (**B**), subscapular (**C**), suprailiac (**D**), supraspinal (**E**), abdominal (**F**), front thigh (**G**), and medial calf (**H**) skinfolds.

**Figure 2:**
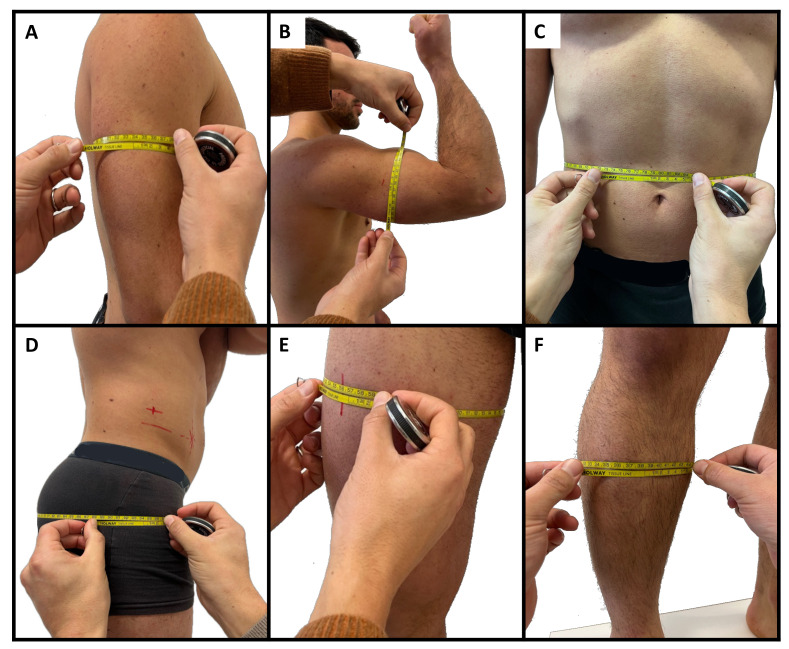
Illustration of the relaxed (**A**) and contracted arm (**B**), waist (**C**), hip (**D**), thigh (**E**), and calf (**F**) circumferences.

**Figure 3:**
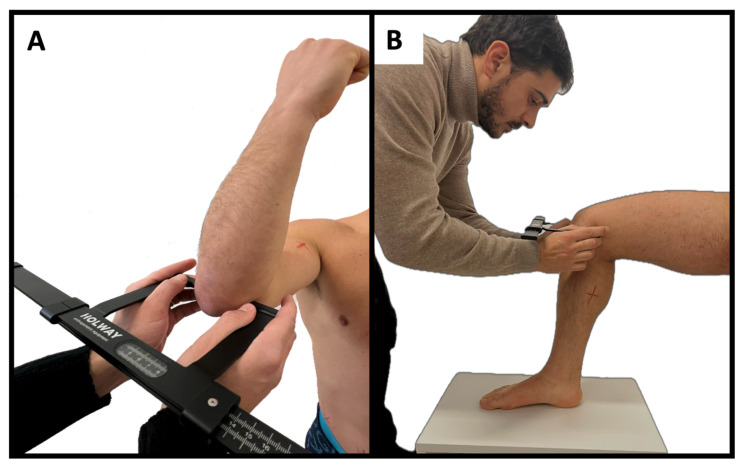
Illustration of the humerus (**A**) and femur (**B**) breadths.

Body mass and stature were measured using a scale with a stadiometer (Seca, Hamburg, Germany) to the nearest 0.1 kg and 0.1 cm, respectively. Body mass index was calculated as body mass (kg)/squared stature (m^2^). Eight skinfold thicknesses (biceps, triceps, subscapular, suprailiac, supraspinal, abdominal, front thigh, and medial calf), six circumferences (relaxed and contracted arm, waist, hip, thigh, and calf), and two bone breadths (humerus and femur) were measured by a level 3 anthropometrist following the procedures established by the ISAK [6]. Figure 1, Figure 2 and Figure 3 illustrate the skinfold, circumference, and breadth measurements, respectively. Skinfold thicknesses were measured to the nearest 0.1 mm using a caliper (Holways, San Jose, CA, USA), circumferences were taken to the nearest 0.1 cm using a measuring tape (Holways, San Jose, CA, USA), and breadths were measured to the nearest 0.1 cm using a sliding caliper (Holways, San Jose, CA, USA). Repeated measures for each parameter were collected to determine the technical error of measurement (TEM) [11]. The equations of Carter and Heath [12] were used to calculate anthropometric somatotypes, as follow:

Endomorphy = −0.7182 + 0.1451 (X) − 0.00068 (X^2^) + 0.0000014 (X^3^), where X = (sum of triceps, subscapular and supraspinal skinfolds) multiplied by (170.18/stature in cm).

Mesomorphy = 0.858 × humerus breadth + 0.601 × femur breadth + 0.188 × corrected arm girth + 0.161 × corrected calf girth − stature × 0.131 + 4.5.

Ectomorphy = if HWR < or equal to 38.25 ectomorphy = 0.1, where HWR = (height divided by the cube root of weight).

If HWR > 38.25 and <40.75 = 0.463 × HWR − 17.63.

If HWR > or equal to 40.75 = 0.732 × HWR − 28.58.

The phantom stratagem [13] was used to calculate Z-scores of each raw variable, as follows:

Z = (x − μ)/σ, where x is the measured variable from the soccer players, μ is the mean value of the general population, and σ its standard deviation. A Z-value = 0.00 indicates that a particular score is proportionally the same as that of the phantom. A positive Z-value indicates that it is larger, and a negative Z-value indicates that it is smaller.

Fat mass, adipose tissue, and skeletal muscle mass were estimated for descriptive purposes only. Fat mass was determined using the equation developed by Peterson et al. [14] and then converted to adipose tissue by multiplying it by 1.18 [15]. Skeletal muscle mass was estimated using the equation proposed by Lee et al. [16].

### 2.3. Statistical Analysis

Data were analyzed with SPSS v. 29.0 (SPSS, IBM Corp., Chicago, IL, USA). The Shapiro–Wilk test was used to check the normal distribution of data. The sphericity of the data was preliminarily assessed using Mauchly’s test. Analyses were performed to complete the following tasks: (i) test whether the anthropometric parameter differed by sex (males vs. females), population (soccer vs. general), and playing position using a one-way analysis of variance (ANOVA) with a Bonferroni post hoc test; (ii) estimate the reference anthropometric percentiles (15th, 50th, and 85th), stratified by sex and playing position. Statistical significance was predetermined as *p* < 0.05. 

## 3. Results

The TEM scores were within 5% for skinfolds and within 1% for circumferences and breadths. The soccer players showed a fat mass percentage of 13.5 ± 2.7 and 25.5 ± 2.9 for males and females, respectively. Fat mass was converted to adipose tissue, yielding 15.9 ± 3.2% for males and 30.1 ± 3.4% for females. The percentage of skeletal muscle mass was 52.5 ± 2.4 and 43.5 ± 3.1 for males and females, respectively. The general participants showed a fat mass percentage of 20.9 ± 3.7 and 30.5 ± 4.6 for males and females, respectively. Fat mass was converted to adipose tissue, yielding 24.6 ± 4.4% for males and 35.9 ± 5.4% for females. The percentage of skeletal muscle mass was 45.8 ± 1.9 and 37.1 ± 1.6 for males and females, respectively. 

**Figure 4:**
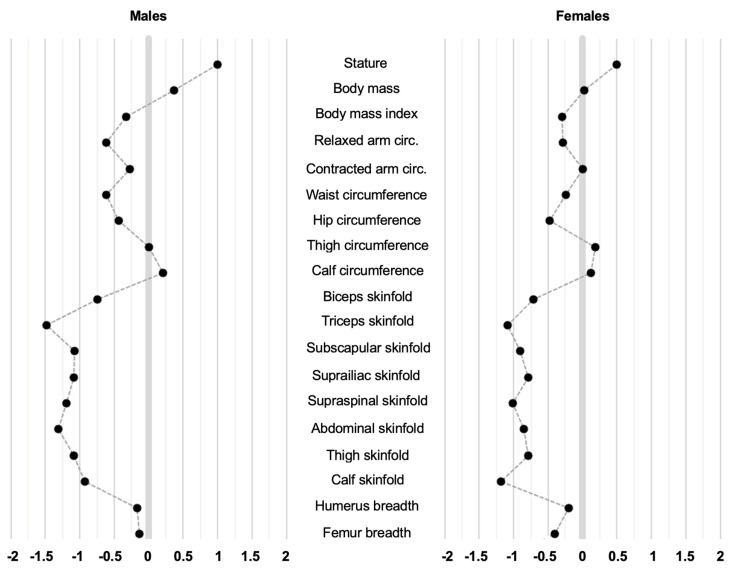
Phantom Z-scores for the soccer players vs. the general population as reference.

Table 1 presents a comparison of the anthropometric characteristics between the soccer players and the individuals from the general population. No differences were found in age among the groups. When considering participants of the same sex, the body mass index was higher for the general population than for soccer players. Circumferences were generally higher for individuals in the general population, except for thigh and calf circumferences, where soccer players had higher measurements. Skinfold measurements were lower for the soccer players. Endomorphy and mesomorphy were higher for the individuals of the general population, whereas ectomorphy was higher for the soccer players. Phantom Z-scores for each anthropometric variable are shown in Figure 4.

The role- and sex-derived reference percentiles for the anthropometric characteristics are reported in Table 2, Table 3, Table 4 and Table 5 for goalkeepers, defenders, midfielders, and forwards, respectively. Data are reported as the mean and the lower and upper bounds of the 95% confidence interval.

Figure 4 shows the somatotype profiles for male and female soccer players sorted by the players’ roles. From the 13 categories proposed by Heath and Carter’s method, the soccer players were found to occupy four somatotype categories (Figure 4). Goalkeepers were mesomorph–ectomorphs and balanced mesomorphs in the cases of males and females, respectively. Defenders were ectomorphic mesomorphs and balanced mesomorphs in the cases of males and females, respectively. Midfielders were ectomorphic mesomorphs and mesomorph–ectomorphs in the cases of males and females, respectively. Forwards were ectomorphic mesomorphs and endomorphic mesomorphs in the cases of males and females, respectively. The individuals from the general population were endomorphic mesomorphs for both sexes (Table 1). One-way ANOVA revealed no difference in somatotype components between players of different roles for either males (endomorphy: F = 0.48, *p* = 0.70; mesomorphy: F = 1.33, *p* = 0.27; ectomorphy: F = 1.43, *p* = 0.24) or females (endomorphy: F = 1.40, *p* = 0.24; mesomorphy: F = 0.79, *p* = 0.50; ectomorphy: F = 2.21, *p* = 0.89). 

## 4. Discussion

The aim of the present study was to provide anthropometric reference values based on ISAK measurement procedures for elite male and female soccer players, categorized by playing position, and to compare them with a group of individuals from the general population. One-way ANOVA was performed, and phantom Z-scores were generated for all raw anthropometric features, demonstrating how the elite soccer player’s profile exhibits distinct characteristics in body composition traits compared to the general population. The analysis of the morphological profile defined by somatotype revealed that adipose-tissue-related characteristics are more pronounced in the general population, whereas soccer players exhibit greater linear dimensions. Lastly, the 15th, 50th, and 85th reference percentiles for all the anthropometric traits were provided for the elite soccer players, stratified by playing position and sex, and can serve as standards for an accurate assessment of anthropometrics-based body composition.

**Figure 5:**
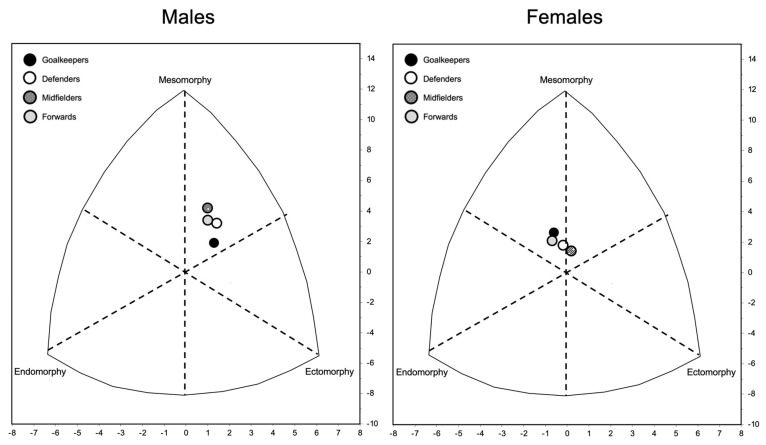
Somatotypes of the soccer players sorted by role and sex.

The anthropometric parameters measured in this study revealed that soccer players were taller than individuals from the general population, while body mass was only higher in males, resulting in a lower body mass index. These differences may be attributed to a reduced fat mass, as indicated by the data concerning the sum of the 8 skinfolds (triceps, subscapular, biceps, suprailiac, supraspinal, abdominal, front thigh, and medial calf), and the estimated body fat, which were lower for soccer players than for the general population. Additionally, body circumferences tended to be larger in the general population; however, considering the generally higher body mass of the soccer players, this may suggest a greater influence of the muscular or skeletal component on body composition, as confirmed by the skeletal muscle mass estimations. In this regard, although the mesomorphic component has been found to be higher in the general population, values ranging from 3 to 5 do not suggest significant differences in morphology, as they fall within a range considered moderate for this somatotype component [12]. Aligning with recent literature [17,18], our results are consistent with the previously reported morphology of elite soccer players, characterized as ectomorphic mesomorphs for males and mesomorphic-endomorphs for females, where physiologically higher amounts of adipose tissue are generally present. Indeed, as can be observed from the somatochart (Figure 5), the distribution of the somatotype of female soccer players shifts towards the lower left quadrant, indicating a higher amount of body fat than males, regardless of their playing position. Conversely, the somatotype of the general population was endomorphic mesomorph regardless of sex, which may be due to less emphasis on non-optimal adipose tissue levels that can negatively impact athletic performance when elevated [8]. As in other sports [19,20], playing positions also play a discriminatory role in body composition, necessitating specific references for both sport and sex as well as playing position. The new references established in this study can, therefore, be applied to soccer players of different genders and positions on the field, allowing an optimal evaluation of body composition, while maintaining the practicality of the anthropometry technique. 

The participants involved in this study were active players in the first Italian soccer league, Serie A. In the literature, various studies have explored the anthropometric characteristics of elite male soccer players [3,21]. However, to the best of our knowledge, this is the first study presenting a comprehensive profile of raw anthropometric measurements (skinfolds, circumferences, and breadths) for female Serie A soccer players. Consequently, comparing these findings to those from female soccer players in other leagues proves challenging. It is well established that body composition, particularly concerning bodily fluids, undergoes changes throughout the season, influenced by both training loads and different phases of the menstrual cycle [22,23,24]. Indeed, elite soccer players, similar to other high-level athletes, possess a distinct body composition profile that may undergo changes throughout the competitive season [25]. Considering the impact of adiposity and lean soft tissue levels on both aerobic and anaerobic performance in elite soccer players, assessing body composition becomes particularly valuable during the preparatory or return-to-play phases [25]. Transition periods are deemed opportune “windows” for achieving significant gains of muscle mass and losses of fat mass, while the rehabilitation process involves monitoring to ensure that body composition features return to their pre-injury state [26,27]. Generally, various tools are used to estimate components related to fat or muscle mass, differing in cost and accuracy [28]. However, when assessing body composition, it is crucial to acknowledge that different assessment methods may yield disparate estimates, irrespective of the considered body mass component [29]. In the context of soccer, the application of various equations and methods often leads to a variation in fat mass values, ranging from 11% to 14% [27,28,29,30]. This variability in the procedures employed makes it challenging and not always reliable to compare values from different studies. Recent research prefers to provide reference parameters for raw measurements to avoid artifacts stemming from theoretical assumptions about body composition or estimation errors due to predictive models, especially in athletes [29,30,31,32]. Moreover, given that laboratory techniques may not always be available, having low-cost and time-efficient tools, such as anthropometry, is essential for practitioners and researchers interested in field evaluations.

This study boasts several strengths, including the careful selection of elite adult male and female soccer players with international experience and a control group of the same age. Furthermore, anthropometric measurements were meticulously gathered in adherence to the ISAK protocol by a certified level 3 anthropometrist, ensuring a high degree of accuracy and precision in the collected data [11]. In situations where logistical or financial constraints are at play, the newly established anthropometric standards offer a valid alternative to more accurate yet less accessible methods, such as dual-energy X-ray absorptiometry, for evaluating body composition. Notably, the cost associated with high-quality materials required for measuring the considered anthropometric dimensions can range between $500 to $1000, exclusive of additional expenses, such as those incurred in bioelectrical impedance analysis, where disposable electrodes are necessary. Regarding time, each subject typically takes between 5 to 10 min to complete an anthropometric assessment, dependent on the complexity and number of measurements involved.

Our study has some limitations. Firstly, our references may not be applicable to lower-level soccer players, given that differences between competitive levels are well documented in the literature [33]. Additionally, we did not assess changes during the season; hence, potential variations may arise when comparing data based on different competitive periods [34]. Furthermore, we did not record the phase of the menstrual cycle for the female participants. Lastly, these references should be exclusively used for adult soccer players to avoid potential effects of maturation on body composition [35,36,37]. Looking into future perspectives, one potential avenue could involve providing anthropometric reference data for the general population, stratified by age and gender categories. Additionally, an intriguing prospect might be the validation of predictive equations for monitoring longitudinal changes during the competitive season and developing specific equations tailored for soccer players to estimate various body mass components. This aspect could be a valuable undertaking, considering the current absence, to our knowledge, of such equations. 

### Future Perspectives

**Figure 6:**
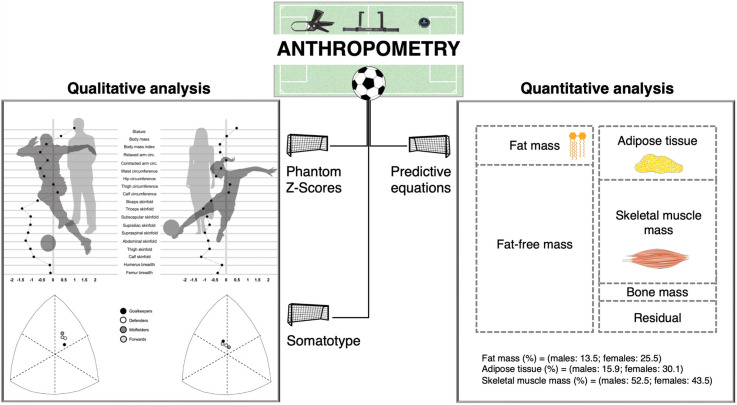
Anthropometrics-based qualitative and quantitative analyses of body composition in soccer players.

Representing a qualitative analysis of body composition, the assessment of raw anthropometric data through phantom Z-scores and somatotype should be encouraged. This approach complements the commonly used quantitative analysis, which involves the utilization of raw measurements within predictive equations for estimating body mass components, such as fat, adipose tissue, and skeletal muscle mass. In this regard, it should be considered that data obtained from quantitative analysis are comparable only when the same predictive equations are used. In addition, it should be considered that fat mass and adipose tissue represent two distinct components, and equations estimating adipose tissue tend to provide higher values than those estimating fat mass [16]. This is because fat mass pertains to the second level of body composition organization, namely, the molecular level. The tissue level, on the other hand, involves a small amount of water combined with other molecules to give rise to more complex and heavier components [16]. Therefore, the analysis of body dimensions or shape based on anthropometric measurements could precede and complement the estimation of body-mass components. Figure 6 schematizes the abovementioned possibilities used in this study for the assessment of body composition in soccer players.

## 5. Conclusions

In conclusion, this study presents anthropometric reference parameters measured according to the ISAK guidelines for elite male and female soccer players. These parameters can serve as benchmarks to establish soccer-specific phantom Z-scores and monitor the body composition of players across different sexes and playing positions. The provided data encompass dimensional measurements such as stature, body mass, circumferences, skinfold thickness, and breadths, along with morphological data related to somatotype. These findings offer a valuable resource for assessing body composition while retaining the practicality associated with anthropometric techniques. The reference values provided in this study contribute to a comprehensive understanding of the anthropometric profile of elite soccer players, facilitating tailored assessments and monitoring within the sport.

## Figures and Tables

**Table 1:** Anthropometric characteristics with means and standard deviations for the soccer players and the individuals from the general population.

	Soccer Players		General Population		One-Way ANOVA
Variable	Males (N = 184)	Females (N = 173)	Males (N = 188)	Females (N = 175)	F, *p*-Value
Age (y)	24.3 ± 4.3	25.2 ± 5.1	24.2 ± 4.8	25.0 ± 5.1	1.7, *p* = 0.16
Stature (m)	183.9 ± 6.2 ^#,§,^^	169.2 ± 6.1 *^,§,^^	177.4 ± 6.5 *^,#,^^	165.8 ± 6.8 *^,#,§^	282.9, *p* < 0.01
Body mass (kg)	79.2 ± 12.1 ^#,§,^^	62.4 ± 6.6 *^,§^	75.9 ± 8.9 *^,#,^^	62.2 ± 8.6 *^,§^	222.1, *p* < 0.01
Body mass index (kg/m^2^)	23.3 ± 1.3 ^#,§,^^	21.8 ± 1.7 *^,§,^^	24.0 ± 2.1 *^,#,^^	22.5 ± 2.3 *^,#,§^	51.4, *p* < 0.01
Circumferences					
Relaxed arm (cm)	29.0 ± 1.8 ^#,§,^^	26.4 ± 1.8 *^,§,^^	30.8 ± 2.9 *^,#,^^	27.1 ± 2.4 *^,#,§^	137.3, *p* < 0.01
Contracted arm (cm)	32.5 ± 1.8 ^#,§,^^	28.0 ± 2.0 *^,§^	33.3 ± 3.0 *^,#,^^	28.0 ± 3.3 *^,§^	212.6, *p* < 0.01
Waist (cm)	75.9 ± 6.4 ^#,§,^^	69.2 ± 3.8 *^,§^	79.7 ± 6.1 *^,#,^^	70.6 ± 5.9 *^,§^	172.2, *p* < 0.01
Hip (cm)	93.4 ± 7.9 ^§,^^	94.0 ± 4.8 ^§,^^	95.8 ± 5.6 *^,#^	97.3 ± 6.9 *^,#^	14.7, *p* < 0.01
Thigh (cm)	54.8 ± 2.8 ^#,^^	53.6 ± 3.6 *^,§^	54.8 ± 4.2 ^#,^^	52.9 ± 3.8 *^,§^	156.8, *p* < 0.01
Calf (cm)	37.5 ± 2.0 ^#,^^	35.9 ± 2.2 *^,§^	37.0 ± 2.3 ^#,^^	35.6 ± 2.6 *^,§^	20.68, *p* < 0.01
Skinfolds (SKF)					
Biceps (mm)	2.9 ± 0.5 ^#,§,^^	4.4 ± 1.7 *^,^^	4.3 ± 1.9 *^,#,^^	6.9 ± 3.5 *^,#,§^	102.0, *p* < 0.01
Triceps (mm)	6.5 ± 2.1 ^#,§,^^	12.6 ± 3.5 *^,§,^^	10.9 ± 2.9 *^,#,^^	19.6 ± 6.4 *^,#,§^	319.8, *p* < 0.01
Subscapular (mm)	7.5 ± 1.3 ^#,§,^^	9.4 ± 2.8 *^,§,^^	12.7 ± 4.8 *^,#,^^	14.7 ± 5.8 *^,#,§^	114.1, *p* < 0.01
Suprailiac (mm)	6.6 ± 2.3 ^#,§,^^	9.8 ± 4.7 *^,§,^^	13.9 ± 6.7 *^,#^	15.4 ± 7.1 *^,#^	92.8, *p* < 0.01
Supraspinal (mm)	6.2 ± 2.1 ^#,§,^^	8.3 ± 3.2 *^,§,^^	13.6 ± 6.2 *^,#,^^	15.5 ± 7.1 *^,#,§^	123.6, *p* < 0.01
Abdominal (mm)	8.7 ± 2.8 ^#,§,^^	13.6 ± 5.3 *^,§,^^	20.9 ± 9.3 *^,#^	20.2 ± 7.8 *^,#^	132.5, *p* < 0.01
Thigh (mm)	8.6 ± 2.9 ^#,§,^^	19.4 ± 5.0 *^,§,^^	15.5 ± 6.3 *^,#,^^	26.1 ± 8.4 *^,#,§^	263.7, *p* < 0.01
Calf (mm)	4.1 ± 1.4 ^#,§,^^	7.8 ± 4.9 *^,^^	8.4 ± 4.6 *^,^^	16.3 ± 7.2 *^,#,§^	186.0, *p* < 0.01
Sum of the 8 SKF (mm)	51.3 ± 9.4 ^#,§,^^	81.8 ± 19.7 *^,§,^^	100.4 ± 31.7 *^,#,^^	133.4 ± 43.7 *^,#,§^	239.8, *p* < 0.01
Breadths					
Humerus (cm)	6.7 ± 0.6 ^#,§,^^	6.1 ± 0.5 *^,§^	6.8 ± 0.6 *^,#,^^	6.2 ± 0.5 *^,§^	74.5, *p* < 0.01
Femur (cm)	9.8 ± 1.0 ^#^	9.3 ± 0.8 *^,§,^^	9.9 ± 0.7 ^#^	9.7 ± 1.0 ^#^	15.3, *p* < 0.01
Somatotype					
Endomorphy	1.8 ± 0.5 ^#,§,^^	3.2 ± 0.9 *^,§,^^	3.6 ± 1.7 *^,#,^^	4.9 ± 1.5 *^,#,§^	154.6, *p* < 0.01
Mesomorphy	3.8 ± 1.5 ^§^	3.3 ± 2.6 ^§,^^	4.9 ± 1.2 *^,#^	4.3 ± 1.3 ^#^	26.6, *p* < 0.01
Ectomorphy	2.8 ± 0.8 ^§,^^	2.7 ± 0.9 ^§,^^	2.2 ± 1.0 *^,#^	2.1 ± 1.0 *^,#^	16.0, *p* < 0.01

Note: * = different from male soccer players; ^#^ = different from female soccer players; ^§^ = different from the general male population; ^^^ = different from the general female population.

**Table 2:** Anthropometric reference percentiles presented as mean and lower and upper bounds of the 95% confidence interval (CI) for the goalkeepers.

	Males (N = 24)			Females (N = 22)		
Variable	15th (95% CI)	50th (95% CI)	85th (95% CI)	15th (95% CI)	50th (95% CI)	85th (95% CI)
Stature (cm)	188.4 (188.0–191.4)	193.0 (190.0–194.0)	194.8 (193.0–195.0)	165.0 (159.0–168.0)	168.5 (167.0–172.0)	176.3 (169.8–180.0)
Body mass (kg)	79.5 (79.2–85.9)	85.9 (80.6–87.6)	91.8 (85.9–92.8)	56.6 (51.8–59.1)	61.3 (57.6–72.1)	72.5 (66.8–72.7)
Circumferences						
Relaxed arm (cm)	28.0 (28.0–30.0)	30.0 (28.0–33.0)	33.0 (31.6–33.0)	24.3 (24.0–26.3)	27.0 (26.0–28.8)	29.0 (28.4–29.5)
Contracted arm (cm)	30.7 (30.5–33.2)	33.2 (31.4–35.9)	36.2 (34.9–36.3)	26.3 (26.0–28.3)	28.8 (27.5–30.3)	30.6 (30.0–31.5)
Waist (cm)	75.0 (75.0–76.6)	79.0 (75.0–79.0)	80.6 (78.4–81.0)	65.0 (63.0–66.5)	67.5 (65.0–70.0)	73.1 (69.6–76.0)
Hip (cm)	92.2 (92.0–93.0)	93.0 (93.0–98.0)	99.6 (94.6–100.0)	90.0 (85.0–92.7)	94.5 (91.0–96.5)	100.7 (95.8–102.1)
Thigh (cm)	48.4 (48.0–55.0)	55.0 (50.0–58.0)	59.6 (55.8–60.0)	50.0 (49.0–52.5)	54.0 (51.0–68.9)	57.2 (55.8–58.0)
Calf (cm)	34.2 (34.0–36.2)	37.0 (35.0–39.0)	39.0 (38.4–39.0)	33.3 (31.0–35.0)	35.0 (34.6–38.0)	39.5 (37.5–40.5)
Skinfolds (SKF)						
Biceps (mm)	2.2 (2.2–2.6)	2.6 (2.4–3.0)	3.3 (2.9–3.4)	3.0 (3.0–3.3)	3.7 (3.2–4.6)	5.0 (4.3–5.0)
Triceps (mm)	4.8 (4.8–5.8)	9.0 (4.8–12.0)	12.8 (9.0–13.0)	9.3 (7.8–10.6)	12.8 (10.0–13.4)	13.9 (13.0–17.0)
Subscapular (mm)	6.4 (6.4–7.1)	7.4 (6.4–10.4)	11.2 (9.5–11.4)	6.3 (5.0–8.0)	8.2 (8.0–9.0)	11.3 (9.0–13.0)
Suprailiac (mm)	3.9 (3.6–5.4)	5.4 (5.0–8.2)	8.8 (7.3–9.0)	6.0 (4.6–6.3)	7.0 (6.0–9.5)	10.8 (8.7–13.0)
Supraspinal (mm)	5.4 (5.2–6.5)	7.0 (6.0–8.0)	12.6 (7.0–13.8)	5.0 (4.6–6.0)	6.5 (5.5–8.5)	11.3 (7.8–13.0)
Abdominal (mm)	6.9 (6.6–8.4)	9.0 (8.0–11.0)	14.2 (9.8–15.0)	6.9 (5.4–8.7)	10.5 (8.1–13.0)	16.3 (12.0–20.0)
Thigh (mm)	6.2 (5.6–11.0)	11.0 (8.6–13.0)	13.8 (12.6–14.0)	11.8 (10.0–16.0)	17.0 (16.0–19.0)	24.8 (17.8–25.0)
Calf (mm)	0.8 (0.3–3.0)	3.0 (2.6–5.0)	6.3 (4.6–6.6)	3.1 (3.0–3.8)	4.0 (3.2–5.0)	5.2 (4.8–12.0)
Sum of the 8 SKF (mm)	39.9 (37.6–53.3)	59.0 (49.2–69.7)	69.9 (65.7–70.0)	58.2 (53.0–64.1)	68.8 (62.4–82.2)	92.8 (78.8–97.5)
Breadths						
Humerus (cm)	6.0 (5.9–7.0)	7.0 (6.4–7.5)	7.9 (7.2–8.0)	5.4 (5.3–5.6)	5.9 (5.6–6.2)	6.5 (6.0–7.0)
Femur (cm)	8.9 (8.8–9.6)	10.0 (9.2–10.4)	10.4 (10.1–10.5)	8.8 (7.0–9.0)	9.4 (9.0–9.8)	10.4 (9.8–10.6)
Somatotype						
Endomorphy	1.1 (1.0–1.5)	2.1 (1.3–2.6)	2.8 (2.3–2.9)	2.1 (1.9–2.6)	2.9 (2.5–3.1)	3.6 (3.0–4.3)
Mesomorphy	1.3 (0.9–3.2)	3.7 (2.9–4.2)	4.3 (3.9–4.3)	1.9 (1.6–3.6)	3.9 (3.3–4.9)	5.3 (4.4–5.5)
Ectomorphy	2.8 (2.8–3.4)	3.4 (2.9–3.6)	3.7 (3.4–3.8)	1.8 (0.9–2.3)	2.6 (2.2–3.2)	3.6 (3.0–3.8)

**Table 3:** Anthropometric reference percentiles presented as mean and lower and upper bounds of the 95% confidence interval (CI) for the defenders.

	Males (N = 52)			Females (N = 50)		
Variable	15th (95% CI)	50th (95% CI)	85th (95% CI)	15th (95% CI)	50th (95% CI)	85th (95% CI)
Stature (cm)	179.0 (170.4–181.0)	186.0 (182.0–188.0)	192.5 (188.0–195.0)	163.1 (161.0–165.0)	168.0 (167.0–171.4)	175.0 (174.0–177.2)
Body mass (kg)	74.2 (69.7–76.6)	79.8 (77.6–83.7)	87.1 (83.8–89.0)	55.6 (54.1–56.5)	61.5 (59.3–63.2)	68.7 (65.9–70.3)
Circumferences						
Relaxed arm (cm)	28.0 (27.0–28.0)	29.0 (28.0–29.0)	31.0 (30.0–33.0)	24.0 (23.0–25.0)	26.0 (25.0–26.0)	27.0 (26.2–28.0)
Contracted arm (cm)	30.7 (30.2–31.2)	31.7 (31.2–32.2)	33.8 (32.7–35.7)	25.4 (24.9–26.5)	27.2 (26.8–27.8)	28.9 (28.1–30.0)
Waist (cm)	74.0 (72.0–75.3)	77.0 (76.0–77.4)	78.5 (78.0–80.0)	65.0 (64.0–66.0)	68.0 (67.0–69.0)	71.4 (71.0–75.2)
Hip (cm)	91.5 (89.0–92.5)	94.0 (93.0–95.0)	96.5 (95.0–98.0)	89.0 (88.6–91.0)	94.0 (92.5–96.0)	98.0 (97.0–100.0)
Thigh (cm)	52.0 (51.0–54.0)	54.0 (54.0–55.0)	57.0 (55.5–58.0)	50.0 (49.4–51.0)	53.5 (52.0–55.0)	56.0 (55.2–58.0)
Calf (cm)	35.5 (34.0–36.0)	38.0 (36.0–38.0)	39.5 (38.5–40.0)	33.9 (33.0–34.2)	36.0 (35.0–37.0)	38.2 (37.5–39.0)
Skinfolds (SKF)						
Biceps (mm)	2.6 (2.6–2.8)	3.0 (2.8–3.0)	3.1 (3.0–3.4)	2.8 (2.4–3.0)	3.4 (3.0–3.8)	5.0 (4.3–6.0)
Triceps (mm)	4.7 (4.0–5.0)	5.4 (5.0–6.0)	6.4 (6.1–8.6)	8.6 (7.8–11.0)	12.0 (11.8–13.0)	15.0 (13.4–16.0)
Subscapular (mm)	6.0 (5.6–6.5)	7.0 (6.6–7.8)	8.1 (7.8–9.6)	7.1 (6.4–7.8)	9.0 (8.2–9.2)	11.0 (10.0–12.0)
Suprailiac (mm)	4.4 (4.0–5.0)	5.6 (5.0–6.6)	8.2 (6.6–11.0)	5.2 (4.8–6.0)	8.0 (7.0–8.2)	10.1 (9.2–14.0)
Supraspinal (mm)	4.8 (4.4–5.4)	6.0 (5.8–7.0)	10.0 (7.0–12.6)	5.5 (5.4–6.5)	8.0 (7.0–9.0)	10.4 (9.8–13.2)
Abdominal (mm)	6.4 (4.0–7.2)	8.0 (7.2–9.6)	10.5 (9.6–15.0)	9.0 (7.5–10.0)	12.0 (12.0–14.2)	18.0 (15.2–19.0)
Thigh (mm)	6.0 (4.4–7.0)	8.0 (7.6–10.0)	11.0 (10.3–13.0)	14.0 (13.2–15.8)	18.0 (17.0–21.0)	24.2 (22.0–28.0)
Calf (mm)	3.1 (3.0–3.3)	3.6 (3.4–3.8)	4.4 (3.9–4.4)	3.0 (3.0–3.2)	5.0 (3.6–5.6)	13.6 (11.0–16.0)
Sum of the 8 SKF (mm)	41.8 (40.4–44.1)	49.0 (44.2–52.4)	57.7 (52.6–61.2)	62.8 (59.4–68.8)	78.2 (72.6–84.2)	98.4 (88.8–103.4)
Breadths						
Humerus (cm)	6.0 (5.5–6.4)	7.0 (6.5–7.2)	7.4 (7.3–7.5)	5.5 (5.3–5.6)	6.0 (6.0–6.2)	6.8 (6.3–7.0)
Femur (cm)	8.9 (6.5–9.0)	9.5 (9.0–10.0)	10.5 (10.0–11.0)	8.6 (8.5–8.9)	9.2 (9.0–9.5)	10.1 (10.0–10.5)
Somatotype						
Endomorphy	1.3 (1.2–1.4)	1.6 (1.4–1.7)	1.9 (1.8–2.4)	2.3 (2.0–2.5)	2.9 (2.8–3.2)	3.5 (3.3–3.8)
Mesomorphy	2.4 (1.0–3.3)	3.9 (3.7–4.3)	5.3 (4.6–5.8)	2.3 (1.6–3.1)	3.9 (3.4–4.4)	5.0 (4.5–5.5)
Ectomorphy	2.1 (1.2–2.4)	3.0 (2.6–3.3)	3.6 (3.3–3.6)	1.8 (1.6–2.3)	2.8 (2.6–2.9)	3.8 (3.2–3.9)

**Table 4:** Anthropometric reference percentiles presented as mean and lower and upper bounds of the 95% confidence interval (CI) for the midfielders.

	Males (N = 53)			Females (N = 43)		
Variable	15th (95% CI)	50th (95% CI)	85th (95% CI)	15th (95% CI)	50th (95% CI)	85th (95% CI)
Stature (cm)	172.9 (170.5–177.7)	179.0 (178.0–184.0)	187.0 (184.1–190.7)	164.7 (158.9–167.2)	171.5 (167.0–173.0)	175.0 (172.7–176.7)
Body mass (kg)	67.8 (63.3–71.9)	74.8 (72.0–78.5)	83.4 (78.6–86.3)	56.7 (55.4–58.7)	60.8 (58.6–62.5)	66.4 (62.1–73.7)
Circumferences						
Relaxed arm (cm)	27.0 (26.9–29.0)	30.0 (29.0–30.0)	31.0 (30.0–32.1)	25.0 (24.0–25.7)	26.0 (25.2–26.0)	26.9 (26.0–29.6)
Contracted arm (cm)	30.2 (29.7–31.8)	32.6 (31.8–32.9)	33.6 (32.9–34.9)	26.0 (25.5–26.5)	27.2 (26.5–27.9)	28.5 (27.8–31.0)
Waist (cm)	70.0 (69.0–75.0)	77.0 (75.0–78.0)	79.0 (78.0–80.4)	65.2 (61.6–67.3)	70.0 (67.0–71.2)	73.4 (71.1–79.3)
Hip (cm)	89.9 (85.9–91.9)	93.0 (92.0–94.0)	95.1 (94.0–101.1)	88.5 (87.5–91.0)	93.0 (90.4–94.3)	97.9 (94.3–102.1)
Thigh (cm)	49.9 (48.0–53.0)	56.0 (53.0–57.0)	58.0 (57.0–58.2)	49.2 (34.9–50.7)	52.0 (50.0–53.0)	55.5 (52.5–59.1)
Calf (cm)	34.0 (33.9–36.0)	38.0 (36.0–39.0)	39.0 (39.0–40.0)	33.9 (32.0–34.3)	35.2 (34.3–36.0)	37.7 (36.0–39.5)
Skinfolds (SKF)						
Biceps (mm)	2.8 (2.8–3.0)	3.0 (3.0–3.4)	3.6 (3.4–3.7)	3.0 (3.0–3.4)	4.0 (3.2–4.4)	5.0 (4.4–6.4)
Triceps (mm)	4.9 (4.7–6.4)	7.0 (6.4–7.6)	9.0 (7.6–11.0)	9.4 (7.3–10.4)	11.0 (7.0–8.5)	14.1 (12.3–19.2)
Subscapular (mm)	5.9 (5.6–6.6)	7.4 (6.6–8.0)	8.3 (8.2–9.4)	6.7 (5.6–7.3)	8.0 (7.0–8.5)	12.1 (8.5–18.8)
Suprailiac (mm)	4.9 (4.2–5.0)	5.6 (5.0–6.4)	7.0 (6.4–12.3)	5.8 (4.8–6.9)	8.2 (6.8–9.0)	15.0 (9.0–25.4)
Supraspinal (mm)	5.0 (4.7–5.9)	7.0 (5.4–8.0)	9.7 (8.1–12.1)	5.2 (4.2–6.0)	7.0 (6.0–8.2)	9.7 (8.2–20.6)
Abdominal (mm)	6.2 (5.1–7.0)	9.2 (8.0–11.0)	12.0 (11.1–13.0)	7.3 (5.9–10.0)	11.0 (10.0–12.0)	19.4 (12.0–32.4)
Thigh (mm)	6.2 (5.1–7.0)	8.6 (7.0–11.0)	12.2 (11.0–14.1)	13.6 (13.0–15.3)	18.5 (15.0–20.0)	22.7 (20.0–26.3)
Calf (mm)	3.2 (3.0–3.6)	3.6 (3.6–4.4)	5.6 (4.3–5.8)	3.7 (3.0–4.4)	6.0 (4.4–9.0)	10.7 (8.7–17.1)
Sum of the 8 SKF (mm)	44.7 (40.9–49.3)	54.6 (49.4–59.0)	62.8 (59.0–68.1)	64.7 (50.3–71.1)	72.0 (71.0–81.7)	97.5 (78.5–150.2)
Breadths						
Humerus (cm)	6.0 (5.5–6.5)	6.9 (6.5–7.0)	7.2 (7.0–7.3)	5.6 (5.4–6.0)	6.1 (6.0–6.2)	6.5 (6.2–6.6)
Femur (cm)	7.5 (6.9–9.0)	9.3 (9.0–10.0)	10.6 (10.0–11.1)	8.7 (7.9–9.0)	9.1 (8.9–9.6)	10.2 (9.6–10.4)
Somatotype						
Endomorphy	1.4 (1.3–1.6)	1.7 (1.6–2.0)	2.2 (2.0–2.7)	2.2 (1.7–2.7)	2.9 (2.7–3.0)	4.2 (3.0–5.9)
Mesomorphy	2.3 (1.4–3.4)	4.3 (3.4–4.9)	5.9 (4.9–5.9)	2.4 (2.0–3.1)	3.5 (3.1–3.9)	4.9 (3.9–5.2)
Ectomorphy	1.8 (1.8–1.8)	2.7 (2.5–2.9)	3.9 (2.9–4.1)	1.7 (1.4–2.7)	3.1 (2.6–3.2)	3.9 (3.2–4.1)

**Table 5:** Anthropometric reference percentiles presented as mean and lower and upper bounds of the 95% confidence interval (CI) for the forwards.

	Males (N = 55)			Females (N = 48)		
Variable	15th (95% CI)	50th (95% CI)	85th (95% CI)	15th (95% CI)	50th (95% CI)	85th (95% CI)
Stature (cm)	177.2 (172.0–183.0)	185.5 (181.5–187.0)	190.4 (186.0–192.0)	163.9 (154.0–166.9)	169.8 (167.5–172.8)	177.2 (173.5–183.0)
Body mass (kg)	75.1 (59.2–76.5)	82.3 (76.5–86.4)	91.4 (84.3–93.6)	55.5 (51.1–61.7)	65.3 (62.3–68.1)	73.9 (68.4–80.2)
Circumferences						
Relaxed arm (cm)	28.0 (25.0–29.6)	30.0 (29.0–32.0)	33.3 (31.0–35.0)	25.0 (24.0–26.0)	27.3 (26.0–28.3)	29.0 (28.5–30.0)
Contracted arm (cm)	30.9 (27.4–32.5)	33.0 (32.0–34.7)	35.9 (34.1–37.8)	26.9 (26.0–27.7)	29.4 (27.8–30.0)	30.8 (30.0–32.3)
Waist (cm)	72.0 (71.0–76.6)	79.0 (75.0–80.0)	81.5 (79.3–84.5)	66.0 (65.0–68.0)	70.0 (69.0–71.8)	74.0 (72.1–76.0)
Hip (cm)	92.0 (87.0–93.5)	95.6 (93.0–98.0)	99.0 (97.2–101.8)	88.7 (8705–91.0)	96.0 (94.0–98.0)	101.6 (99.1–106.4)
Thigh (cm)	54.0 (51.0–54.6)	56.5 (54.0–58.0)	60.5 (57.0–63.5)	49.7 (48.0–52.1)	55.0 (53.0–56.0)	58.0 (56.1–60.1)
Calf (cm)	36.0 (34.0–36.)	38.0 (36.0–39.0)	39.4 (38.5–44.0)	34.0 (32.0–35.0)	36.3 (35.0–37.0)	39.0 (37.1–39.0)
Skinfolds (SKF)						
Biceps (mm)	2.9 (2.4–3.0)	3.0 (3.0–3.1)	3.4 (3.0–4.4)	3.0 (2.6–3.0)	3.4 (3.0–3.8)	4.5 (4.0–8.0)
Triceps (mm)	5.1 (4.4–5.6)	6.0 (5.4–6.6)	8.8 (6.5–10.0)	8.0 (7.0–10.8)	14.0 (11.0–15.5)	18.1 (16.0–20.0)
Subscapular (mm)	5.8 (5.0–6.8)	7.4 (6.4–8.1)	9.5 (7.8–11.0)	6.6 (6.4–7.8)	10.1 (8.0–12.7)	14.2 (13.0–15.0)
Suprailiac (mm)	4.2 (3.6–6.0)	6.9 (5.6–8.2)	9.8 (7.5–11.0)	4.9 (4.6–6.6)	8.0 (6.8–9.2)	13.2 (10.0–24.0)
Supraspinal (mm)	4.7 (4.4–5.9)	6.7 (5.4–8.6)	9.2 (8.2–10.0)	5.4 (4.8–6.4)	8.5 (7.2–10.0)	12.1 (10.1–17.0)
Abdominal (mm)	6.1 (5.5–9.0)	10.0 (8.0–11.2)	12.7 (11.0–13.0)	7.2 (5.8–10.9)	13.0 (11.0–15.5)	20.1 (16.0–23.5)
Thigh (mm)	6.2 (5.1–7.0)	8.4 (6.2–9.0)	13.5 (9.0–15.6)	13.9 (9.8–17.0)	20.0 (18.1–22.0)	25.1 (23.0–28.0)
Calf (mm)	5.3 (4.2–6.6)	3.4 (3.2–4.0)	5.1 (3.8–7.0)	3.0 (3.0–4.6)	4.1 (3.8–6.6)	13.5 (7.8–16.0)
Sum of the 8 SKF (mm)	43.2 (38.9–46.5)	51.0 (44.8–60.2)	66.8 (58.2–71.6)	63.8 (49.4–72.4)	86.1 (79.8–95.4)	108.3 (96.1–131.0)
Breadths						
Humerus (cm)	6.2 (6.0–6.7)	7.0 (6.5–7.0)	7.2 (7.0–7.5)	5.5 (5.4–5.8)	6.0 (5.9–6.4)	6.5 (6.4–6.8)
Femur (cm)	8.3 (6.7–9.2)	9.7 (9.0–10.4)	10.7 (10.2–11.2)	8.5 (8.0–9.0)	9.6 (9.1–10.0)	10.4 (10.0–10.8)
Somatotype						
Endomorphy	1.3 (1.0–1.6)	1.9 (1.6–2.1)	2.4 (2.0–2.6)	2.1 (1.7–2.7)	3.3 (2.7–3.9)	4.7 (3.9–5.1)
Mesomorphy	2.0 (1.1–3.3)	4.1 (2.9–5.2)	5.9 (4.9–7.2)	2.9 (2.6–3.6)	4.1 (3.7–2.3)	4.9 (4.5–5.6)
Ectomorphy	1.5 (1.1–2.1)	2.9 (1.9–3.4)	3.7 (3.3–3.9)	1.4 (1.2–1.9)	2.4 (2.0–2.7)	3.3 (2.7–3.8)

## Data Availability

Data can be obtained from Francesco Campa at francesco.campa3@unibo.it.
